# Muscle transcriptome analysis reveals genes and metabolic pathways related to mineral concentration in *Bos indicus*

**DOI:** 10.1038/s41598-019-49089-x

**Published:** 2019-09-03

**Authors:** Juliana Afonso, Luiz Lehmann Coutinho, Polyana Cristine Tizioto, Wellison Jarles da Silva Diniz, Andressa Oliveira de Lima, Marina Ibelli Pereira Rocha, Carlos Eduardo Buss, Bruno Gabriel Nascimento Andrade, Otávio Piaya, Juliana Virginio da Silva, Laura Albuquerque Lins, Caio Fernando Gromboni, Ana Rita Araújo Nogueira, Marina Rufino Salinas Fortes, Gerson Barreto Mourao, Luciana Correia de Almeida Regitano

**Affiliations:** 1 Department of Evolutionary Genetics and Molecular Biology, Federal University of São Carlos, São Carlos, Brazil; 2 Department of Animal Science, University of São Paulo/ESALQ, Piracicaba, Brazil; 3 Embrapa Pecuária Sudeste, São Carlos, Brazil; 4 Physics Institute of São Carlos, University of São Paulo, São Carlos, Brazil; 5 Animal Science department, Laboratory of Molecular Genetics. São Paulo State University, Jaboticabal, Brazil; 6 Bahia Federal Institute of Education, Science and Technology, Ilhéus, Brazil; 7 School of Chemistry and Molecular Biosciences, Faculty of Sciences, The University of Queensland, Brisbane, Australia; 8 NGS Soluções Genômicas, Rua Ajudante Albano 847, Piracicaba, SP Brazil

**Keywords:** Metals, Gene expression, Genomics

## Abstract

Mineral content affects the biological processes underlying beef quality. Muscle mineral concentration depends not only on intake-outtake balance and muscle type, but also on age, environment, breed, and genetic factors. To unveil the genetic factors involved in muscle mineral concentration, we applied a pairwise differential gene expression analysis in groups of Nelore steers genetically divergent for nine different mineral concentrations. Here, based on significant expression differences between contrasting groups, we presented candidate genes for the genetic regulation of mineral concentration in muscle. Functional enrichment and protein-protein interaction network analyses were carried out to search for gene regulatory processes concerning each mineral. The core genetic regulation for all minerals studied, except Zn, seems to rest on interactions between components of the extracellular matrix. Regulation of adipogenesis-related pathways was also significant in our results. Antagonistic patterns of gene expression for fatty acid metabolism-related genes may explain the Cu and Zn antagonistic effect on fatty acid accumulation. Our results shed light on the role of these minerals on cell function.

## Introduction

The role of minerals in meat quality traits is perceived in the nutritional value of beef. For example, high iron content is a major player in the claims regarding nutritional value^1^. The second meat quality trait affected by minerals is beef tenderness since calcium related-proteases take part in the *post-mortem* degradation of myofibrillar proteins^2^. As minerals are essential for a variety of biological processes such as metabolism, homeostasis maintenance, growth, influencing cellular structural components, enzyme cofactors, regulation of cell replication, and differentiation^3^, they may affect other economically important traits in livestock production.

Mineral concentration in mammalian muscles depends on animal intake-outtake imbalance^4^, muscle type^5^, age, environment^6^, breed^7^, and other genetic factors^8^. Minerals can only perform their biological function in muscle cells if they are available in the right amount^3^. Their concentration is under strict control for homeostasis maintenance. The genetic control of mineral homeostasis is not fully understood, although there is evidence regarding specific genes and certain gene functions linked to mineral concentration in many species. Calcium concentration in humans depends upon a complex network of hormones^9^. Both serum and urinary calcium were deemed continuous heritable traits, in a study with twins^9^. Genes related to sodium and potassium homeostasis in humans were reviewed elsewere^10^. Magnesium absorption in bovine is breed dependent^11^. Zinc homeostasis is poorly understood, but in *C*. *elegans* there is a conserved motif called low zinc activation element in promoters that seems to be involved in the process^12^. Genes associated to copper transport in higher eukaryotes (hCTR1/2, Atox1 and Atp7A/B) were detected in a yeast functional screen that aimed to find genes linked to copper-dependent respiratory growth, which could be candidate markers to human mitochondrial diseases^13^. Genes related to iron concentration participate in lipid metabolism in Nelore cattle^14^. Still, information about genes associated with mineral composition in beef is scarce.

Scientific evidence about genetic mechanisms associated with bovine mineral deposition regulation in muscle comes from a limited number of studies. Quantitative trait loci (QTLs) related to mineral concentration were described in Jersey x Limousin crosses^4^, Nelore^15^, Holstein, and Jersey^16^ cattle. These studies reported some overlapping QTLs and enriched functional processes among different minerals, indicating shared genetic regulation.

Once minerals participate in a variety of biochemical processes that might affect production traits, understanding the genetic and physiological processes underlying muscle mineral concentration might provide the basis for modulating these processes to the benefit of cattle production. Selective breeding could incorporate gene polymorphisms that influence mineral composition to improve the nutritional value of beef^17^. Understanding the genetics and gene regulation associated with muscle minerals in cattle may also provide some evidence for how conserved these are across species. Given biochemical similarities across mammals, it is possible that increased knowledge from cattle studies might be generalized to humans.

Herein, to characterize the biological pathways involved in muscle mineral deposition, we described a differential expression RNA-seq analysis from *Longissimus thoracis* muscle of contrasting mineral content Nelore steers, pinpointing genes, processes, and pathways related to mineral homeostasis. The minerals analyzed were Calcium (Ca), Copper (Cu), Potassium (K), Magnesium (Mg), Sodium (Na), Phosphorus (P), Sulfur (S), Selenium (Se), and Zinc (Zn).

## Results

### Animals and RNA-Seq analysis

Each contrasting group for a specific mineral was called Low (L) or High (H) and differentiated by the corresponding mineral symbol. Due to the overlapping of samples among groups, 44 samples comprised our 18 groups. The average GEBV^15^ and mineral concentration for contrasting groups confirmed they were significantly different and comparable, as shown in Table 1. The average number of read pairs aligned was 13,333,842, and the average percentage of reads aligned to the reference Bovine Genome (UMD 3.1) was 91.82%. Our sequencing allowed the identification of a significant number of transcripts. The transcripts discovery saturation curves (discovered transcripts *versus* reads sequenced) from the samples assessed here are shown in Supplementary Fig. S1.

**Table 1:** Statistics of the genetic estimated breeding values and RNA-Seq for each extreme group.

Group	GEBV	St. Error^a^	Concentration^b^	St. Error^c^	Read aligned pairs	Alignment (%)	T-test p-value^d^
Low-Ca	−0.1122	0.007	85.75	9.1261	10,103,844	93.98	
High-Ca	0.1366	0.0247	346.71	31.3709	16,664,657	91.48	8.55E-05
Low-Cu	−0.0607	0.0015	1.13	0.0396	10,280,771	92.9	
High-Cu	0.1228	0.0378	4.46	1.6121	12,204,273	92.15	4.70E-03
Low-K	−0.0447	0.0035	976.05	26.2935	14,682,381	91.22	
High-K	0.0872	0.0028	2152.36	65.5082	13,526,576	91.63	1.30E-10
Low-Mg	−0.0435	0.0026	668.6	14.788	14,682,381	91.22	
High-Mg	0.0773	0.0038	1401.05	32.1704	15,112,850	91.67	1.05E-09
Low-Na	−0.0478	0.0035	1544.6	57.8293	13,098,861	91.57	
High-Na	0.097	0.0047	3807.57	136.1052	15,112,850	91.67	1.05E-09
Low-P	−0.0459	0.002	6354.98	232.1688	13,827,066	91.68	
High-P	0.0843	0.0031	14128.47	523.2822	13,526,576	91.63	1.30E-10
Low-S	−0.061	0.0017	4650.32	260.7678	11,665,773	92.07	
High-S	0.0832	0.0041	11783.28	588.4472	14,037,706	91.23	1.63E-08
Low-Se	−0.1703	0.0087	0.0765	0.0052	12,902,391	91.97	
High-Se	0.1143	0.0066	0.32	0.0209	10,678,101	91.05	5.02E-10
Low-Zn	−0.071	0.0058	58.59	2.6137	13,370,593	91.98	
High-Zn	0.1115	0.0061	183.17	9.4696	14,531,511	91.63	1.04E-09

All values presented are averages of the values inside each extreme group.

^a^standard error of the media for GEBV of each mineral, ^b^average mineral concentration in mg/Kg for each extreme group, ^c^standard error of the media for the mineral concentration of each mineral, ^d^p-value of the test of significance (t-test) between the extreme group samples’ GEBVs for each mineral.

We identified 29,312 transcripts but tested only 15,012 for differential expression due to their expression levels, since Cuffdiff v2.2.1^18^ parameters were set to do not take into account genes with less than ten reads aligned, in both differential expression analysis and multiple test correction.

### Differentially expressed genes (DEGs)

We identified 327 annotated DEGs considering all minerals. The number and annotation status of the DEGs were variable among the evaluated groups (Table 2). All DEGs and their fold change (FC) values between contrasting groups for each mineral are in Supplementary Table S1. There were no common DEGs to all minerals. However, 27genes were common to at least five minerals. From these, we can highlight *COL11A1*, *COMP* and *TNMD* genes, common to eight minerals (all, except Zn). The minerals with more DEGs overlapping were Mg, Na, K, and P, with 25. In all expression comparisons between contrasting groups, upregulation means higher expression in H-groups than in L-groups. Conversely, downregulation means lower expression in H-groups than in L-groups. Unlike Zn, which had 50% of the DEGs downregulated, the remaining minerals presented at least 66% of the DEGs as downregulated.

**Table 2:** Number and Annotation status of DEGs per mineral.

Mineral	Ca	Cu	P	Mg	K	Se	Na	Zn	S
Annotated genes^a^	170	125	43	53	51	25	55	27	15
Predicted proteins^b^	24	7	6	5	5	6	5	4	3
Non-annotated genes^c^	35	23	17	22	23	6	13	4	4
Upregulated^d^	34	13	8	10	9	9	13	15	6
Downregulated^e^	160	119	41	48	47	22	47	16	12
Total	229	155	66	80	79	37	73	35	22

^a^Genes with known annotation based on the bovine reference genome (UMD 3.1), ^**b**^transcripts with predicted annotation, ^**c**^transcripts with unknown annotation, ^**d**^annotated genes and predicted proteins more expressed in the high groups, ^**e**^annotated genes and predicted proteins more expressed in the low groups.

DEGs with the highest estimated FC ( > 1.9) between each contrasting mineral group were *MT2A* for K, Mg, Na, and P; *RN5-8S1* for Se and S; *HSPA6* for Cu, Zn, P and Se; *PMP2* for Cu, and *GBP4* for Ca. DEGs with lowest FC ( < 1.9) between groups were *TNMD*, *COMP*, and *COL11A1* for eight minerals (except for Zn); *FBLN7* in seven of them (except for S and Zn), and *CILP2* in six (except for S, Zn, and Na). Among DEGs with the lowest FC (downregulated in the H-group) for at least two minerals, we found *PERP* for Cu, K, Mg, P, and Se; *TNC* and *THBS4* for Cu, K, Mg, and P; *COL22A1* for Cu, Na, P, and Se; *ADAM12* for Cu and Se; *ACTC1* for *K* and *P*; *CRABP2* and *CRTAC1* for K, Mg, and P; *KCNK2*, *MKX* and *MXRA5* for Ca and Cu.

Regarding individual minerals, we found *TF*, *HOXA9*, *MIR196B*, and *SYT4* genes as top downregulated in H-Ca group; *ELOVL6*, *PTGIR*, *COL12A1*, *GAS2*, *POSTN*, *WISP1*, *MLLT11*, and *THRSP* for Cu; *PI1S for* Na; *MYLK3* for S; and *RN5-8S1* for Zn. From Se and S analyses, *RN5851* gene was upregulated in higher mineral concentration group.

### Functional enrichment analysis

We performed a functional annotation analysis applying the Trinotate pipeline (http://trinotate.sourceforge.net/) to identify possible biological functions of non-annotated DEGs. We retrieved possible functions for 31 transcripts. From these, 18 presented functions related to LINE-1 retrotransposable elements, retrovirus-related Pol polyprotein, and immune response related functions. We also recovered the function “similar to the protein SAMHD1”, a restriction nuclease that suppresses LINE-1 retrotransposition activity^19^ (Supplementary Table S2). Among the non-annotated transcripts from Cu DEGs, one is highly similar to a myoregulin (GO: 0016021), with high homology to a human *MRLN* gene (91.30% of similarity). Another one was annotated as the Sentrin-specific protease 3, having homology with a mouse *SENP3* gene (92.86% of similarity).

We clustered annotated functions obtained with DAVID software^20^ for each predicted protein whose coding gene was a DEG for each mineral. The summarized significant analysis is presented in Table 3. We did not obtain substantial annotated function clusters for Zn and S. Common functions in at least four minerals were related to the extracellular matrix (enriched in seven minerals), extracellular matrix-receptor interaction (ECM-receptor interaction), collagen and secretion, the latest three enriched in six minerals. Also, for five minerals we identified disulfide bond, epidermal growth factor-like domain, focal adhesion and, for four minerals, protein digestion and absorption.

**Table 3:** DEGs summarized significative annotated function clusters.

Ca	Cu	P	Mg	K	Se	Na
Cell-cell interaction	Calcium ion binding	Cell adhesion	Carboxypeptidase	Collagen	Extracellular matrix	Carboxypeptidase
Collagen	Carboxypeptidase	Collagen	Collagen	Disulfide bond		Cell adhesion
ECM-receptor interaction	Cell adhesion	Disulfide bond	Disulfide bond	ECM-receptor interaction		Collagen
Extracellular matrix	Cell-cell interaction	ECM-receptor interaction	ECM-receptor interaction	Epidermal growth factor-like domain		Disulfide bond
Protein digestion and absorption	Collagen	Epidermal growth factor-like domain	Epidermal growth factor-like domain	Extracellular matrix		ECM-receptor interaction
Proteoglycans	Disulfide bond	Extracellular matrix	Extracellular matrix	Focal adhesion		Epidermal growth factor-like domain
Secretion	ECM-receptor interaction	Focal adhesion	Focal adhesion	Glycoprotein		Extracellular matrix
Signaling	Epidermal growth factor-like domain	PI3K-Akt signaling pathway	Glycoprotein	Immunoglobulin-like domain		Focal adhesion
	Extracellular matrix	Protein digestion and absorption	Secretion	Secretion		Glycoprotein
	Fatty acid metabolism	Secretion				Leucine-rich repeat
	Focal adhesion					Protein digestion and absorption
	PI3K-Akt signaling pathway					Secretion
	Protein digestion and absorption					
	Secretion					
	Signaling					

The results were obtained using DAVID software. There are no significative results for Zn e S. Results are displayed for each mineral and in alphabetic order.

### Relationship among minerals

The GEBVs for most mineral concentrations showed significant Pearson correlations in our population, ranging from −0.2 to 0.97 (Supplementary Table S3). Also, high significant correlations were observed between each GEBV and their correspondent raw mineral concentrations, varying from 0.77 to 0.86 (Supplementary Table S4). Results of t-tests to verify if the mean GEBVs of the samples used to represent the contrasting groups for one mineral would also be statistically different for any other mineral are shown in Supplementary Table S5.

### Protein-protein interaction and pathways among DEGs

**Figure 1:**
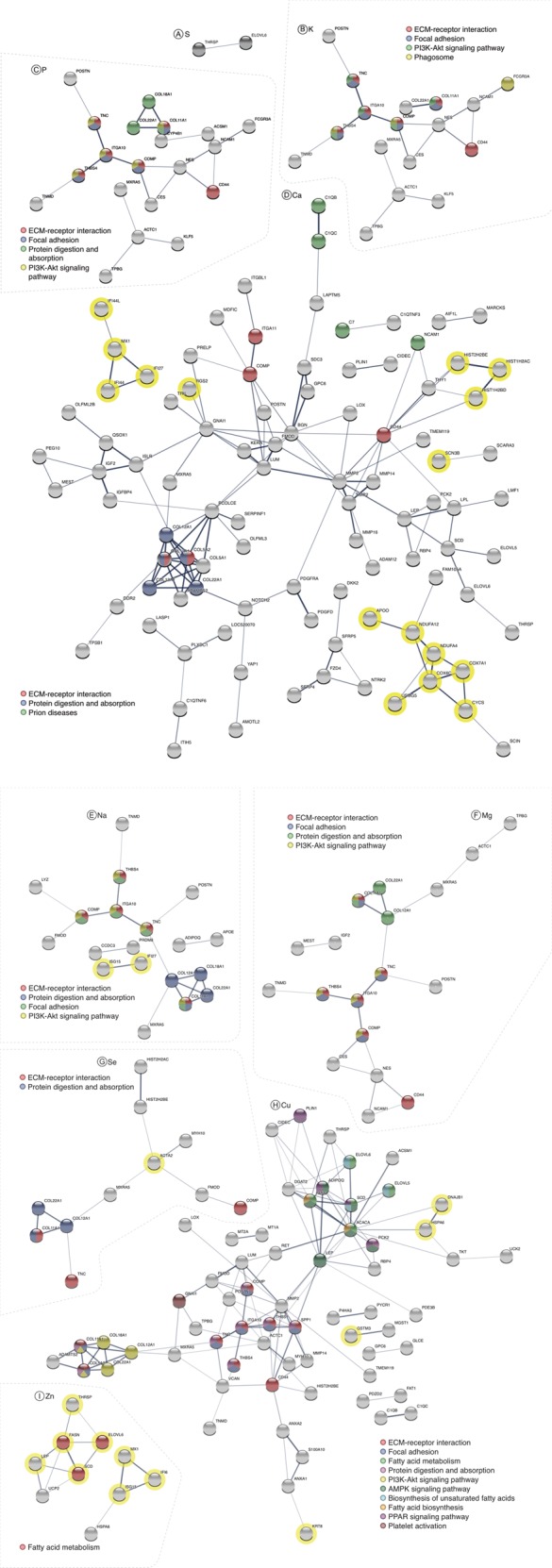
DEGs’ products protein-protein interaction network for each mineral. Proteins not partaking in an interaction are not shown. The line thickness between two proteins indicates the strength of data support. The colors inside the circles represent DEGs participating in the same pathway. The yellow halos represent DEGs upregulated in the H groups in relation to the L groups. The DEGs without a yellow halo were downregulated in the H groups in relation to L groups. (**A**) S, (**B**) K, (**C**) P, (**D**) Ca, (**E**) Na, (**F**) Mg, (**G**) Se, (**H**) Cu, and (**I**) Zn.

To identify biological processes involving the DEGs, we performed a protein-protein interaction (PPI) network analysis among DEGs for each mineral using STRING v.1.2.2 software^18^, which retrieves pathways from KEGG database^21^. DEGs for each mineral partaking in known PPI, and its significant pathways, are shown in Fig. 1. Sulfur did not present a significant pathway.

**Figure 2:**
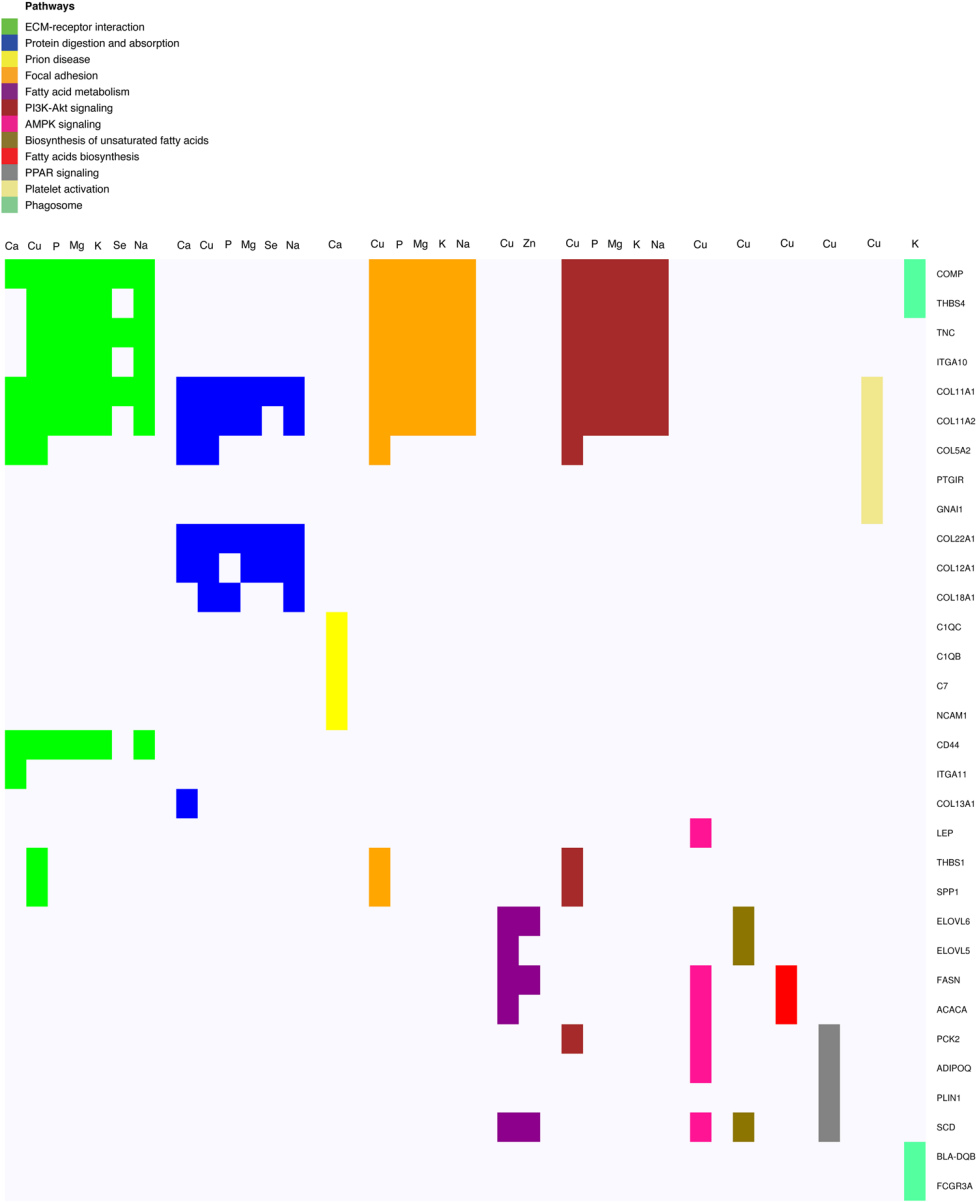
DEGs partaking in significant pathways. Rows: DEGs partaking in the significant pathways, columns: minerals presenting the significant pathways, colors: different significant pathways.

All DEGs presented in the same pathway for a given mineral had the same direction of expression, *i*.*e*., they were either all upregulated or all downregulated (Supplementary Table S1). DEGs presented in each pathway across mineral analyses can be seen in Fig. 2. Significant pathways for all minerals are shown in Table 4. ECM-receptor interaction pathway was common to seven minerals (except Zn and S), protein digestion and absorption pathway was common to six (except Zn, S, and K), and focal adhesion pathway and PI3K-Akt signaling pathway to five (except Ca, Se, Zn, and S). All DEGs presented in these pathways were downregulated.

**Table 4:** DEGs significative KEGG Pathways enriched for each mineral.

KEGG Pathways	Ca	Cu	P	Mg	K	Se	Na	Zn
ECM-receptor interaction	0, 012	2.85e-08	1.45e-07	5.65e-07	4.27e-07	0.0229	9.57e-07	—
Protein digestion and absorption	0, 012	0.0009	0.0028	0.0060	—	0.0229	0.0005	—
Focal adhesion	—	0.0006	0.0006	0.0019	0.0015	—	0.002	—
PI3K-Akt signaling pathway	—	0.0024	0.0053	0.0153	0.0165	—	0.023	—
Fatty acid metabolism	—	0.0009	—	—	—	—	—	0.0104
AMPK signaling pathway	—	0.0056	—	—	—	—	—	—
Biosynthesis of unsaturated fatty acids	—	0.0121	—	—	—	—	—	—
Fatty acid biosynthesis	—	0.0206	—	—	—	—	—	—
PPAR signaling pathway	—	0.0283	—	—	—	—	—	—
Platelet activation	—	0.0311	—	—	—	—	—	—
Phagosome	—	—	—	—	0.0421	—	—	—
Prion diseases	0, 014	—	—	—	—	—	—	—

Sulfur do not present a significative KEGG pathway. P-values displayed for each pathway.

Fatty acid metabolism pathway was common to Zn and Cu. This was the only pathway where DEGs had different regulation between both minerals. Of all DEGs in this pathway, three were common for both minerals (*ELOVL6*, *FASN*, and *SCD*) and two were exclusive to Cu concentration analysis (*ELOVL5* and *ACACA*) (Fig. 2). From all minerals, Cu retrieved more enriched pathways (N = 10), whereas prion disease and phagosome pathways were identified only in Ca and K analyses, respectively.

After filtering out DEGs that did not interact in our PPI network, 96 remained for Ca, 64 for Cu, 17 for K, 18 for Mg, 19 for Na, 20 for P, two for S, 11 for Se, and 10 for Zn. In total, Ca and Cu had more than 50% of their DEGs taking part in an interaction (56.47% and 51.2%, respectively), P and Se had around 45% (46.5% and 44%, respectively), K, Na, Mg, and Zn had about 35% (33.3%, 34.5%, 33.96%, and 37%,) and S had the lowest rate of DEGs in interactions, 13.3%. From DEGs’ products that did not take part in protein-protein interactions, only five were part of a pathway: *COL11A2* for K, Ca, Na, Mg and Cu; *COL11A1* for P; *CILP2* for K; *CD44* for Na; and *PTGIR* for Cu.

## Discussion

### Heritability, GEBVs, and correlations for mineral concentration

Muscle mineral concentrations are moderately heritable traits. Estimates of heritability from our Nelore population ranged from 0.29 to 0.33^15^. Understanding the genetic component related to muscle mineral concentration might be useful to better comprehend mineral metabolism and metabolic diseases.

As expected from the correlations among GEBVs for the minerals and from the biological interconnection among them, most contrasting groups for each mineral also differed concerning other mineral’s GEBVs, except for Cu and Se, even though they did not meet the criteria of representing both 5% extremes from the normal distribution. The most extreme example comes from the minerals Mg, Na, K, and P, presenting a correlation higher than 0.88 among their GEBVs. As a consequence, the same samples comprised the low-Mg and low-K, the high-Mg and high-Na and in the high-K and high-P groups. As the complementary contrasting group for each mineral had at least one different sample, the DEGs, functions, and pathways are not entirely the same among these minerals. Given this fact, one should consider that some correlated response regarding other than the mineral in discussion could exist within our results.

Mg and K, which in our analysis showed a correlation of 0.97 and 46 DEGs in common, presented the same QTLs in previous experiments with this population, thus reinforcing the common genetic control for these minerals. However, Mg and P showed the same pairwise correlation and presented 38 common DEGs, but did not showed QTLs in common^15^. Similarly, despite the correlations, there were no common QTLs among Mg, K, Na, and P^15^. Thus, although not fully explained by pairwise correlation, to some extent, the common DEGs, functions, and pathways among these four minerals can result from the high correlation and sample overlapping among them.

### Detected functions and previous works

All the enriched gene functions convey to one or more enriched pathways. The previous detection of similar functional gene clusters in a GWAS for 14 minerals^15^, whose dataset included our samples, indicates conserved mechanisms affecting mineral concentration. The involvement of common DEGs in shared pathways among minerals reinforces that various genes affect these phenotypes.

Differential expression and QTL analyses can produce similar functional annotation results, but different gene lists due to differences in the methodologies^22^. Our DEGs were not harbored in/or near QTL regions already reported^15^. However, the functional analyses of DEGs and QTLs pointed to similar gene functions. Due to this fact, we will focus our discussion on the genetic similarities among different mineral analyses.

### DEGs with opposite FC among minerals

*MT2A* and *HSPA6* were the DEGs with the expression pattern presenting the highest FC contrariety and discrepancy among minerals. Both genes have a known relationship with heavy metals. The *MT2A* gene encodes a metallothionein protein that binds divalent heavy metals and participates in metal control and Zn homeostasis in the cell, affecting apoptotic and autophagy pathways^23^. *MT2A* was a DEG for almost all minerals in this study, except Ca and Se. It is downregulated in the H-Cu group and upregulated in the H-groups of Mg, P, Zn, Na, S, and K. From these, only Cu and Zn are divalent heavy metals. Different polymorphisms in *MT2A* or its promoter disturb Zn and Cd concentrations in human blood of healthy patients^24^ and carotid artery stenosis patients^25^. Our results suggest that, apart from the already described in the literature, this gene could also be related to the concentration of other non-heavy metal minerals like Mg, K, Na, P, and S.

The *HSPA6* gene was upregulated in the H-Cu and H-S groups, while the opposite occurs for Zn and P. This gene product responds to stress, and its expression increases with the increase of heavy metal, like Cu, concentration^26^. This protein takes part in the fatty acid metabolism pathway (FAM), where Zn and Cu are essential. The relationship between *HSPA6*, P, and S is still unknown.

### Extracellular matrix interactions

Among the downregulated DEGs with the lowest FC accross minerals, lower than −1.9, we found *COMP*, *COL11A1*, *TNC*, *THBS4*, and *COL22A*. They were involved in common pathways for at least six minerals, which may indicate a potential common genetic regulation of mineral concentration or a possible role of mineral concentration in the control of these genes expression. They genes act in pathways such as ECM-receptor interaction, focal adhesion, PI3K-Akt signaling pathway, and protein digestion and absorption. The first three pathways are interconnected (http://www.genome.jp/kegg/pathway/hsa/hsa04510.html).

The DEGs *COMP* and *COL11A1*, common to eight minerals, are part of the ECM-receptor pathway. They encode cell membrane proteins that mediate the interaction between the cell and extracellular matrix^27,28^. Ligands such as *COL11A1* and *COMP* are essential for the initial steps of the ECM-receptor interaction pathway. Integrins continue the pathway processes, culminating in different cell functions such as growth and regeneration^29^. Also, focal adhesion and PI3K-Akt signaling pathways specifically need the involvement of integrins to start their metabolic processes^29^.

The integrin gene *ITGA10* was predicted to interact with *COL11A1* and *COMP*. All analyses showing *COL11A1*, *COMP*, and *ITGA10* also showed the *TNMD* gene. This gene possibly interacts with *ITGA10* by the *THBS4* gene, which is also part of the three connected pathways. Moreover, *ITGA10* connects to *TNC*, involved in collagen formation. Thus, *COL11A1*, *COMP*, and *TNMD* take part in the three integrated pathways for K, P, Na, Mg, and Cu by its interaction with *ITGA10*. Their downregulation in H-groups suggests that a high concentration of these minerals suppresses these pathways.

The ECM-receptor interaction pathway plays an essential role in skeletal muscle development^30^, which explains this pathway being found in muscle transcriptome. Simple diffusion of minerals can occur through pores in the tight junctions if the electrochemical gradient exists to push the ions through the pores^31^. The *CD44* gene, a DEG for almost all minerals, has a possible role in tight junction regulation^32^. The relation of ECM-receptor interaction pathway to mineral concentration may be partially explained by the tight junctions’ role in mineral absorption.

The protein digestion and absorption pathway was significant for six minerals (Ca, Cu, P, Mg, Na, and Se). The DEGs in this pathway encompass genes from the collagen family. Collagens are the most abundant protein in the ECM and take part in cell adhesion regulation, cell migration, and direct tissue development, the latest initiating after modifications in the ECM structure mediated by substrates^6^. These results indicate that ECM-interactions are related to mineral concentration regulation for most of the minerals in this study.

### Zn and Cu antagonism on fatty acid metabolism

Fatty acid metabolism pathway (FAM) was enriched in Zn and Cu analyses. Cu analysis identified five DEGs in this pathway, *ACACA*, *FASN*, *SCD*, *ELOVL6* and *ELOVL5*. From these, *FASN*, *SCD* and *ELOVL6* were the only genes for Zn content in the same pathway. They all showed interactions between their encoded proteins. All DEGs included in this pathway were downregulated in Cu and upregulated in Zn analyses.

Animals with clinical Cu deficiency tend to accumulate fat due to disturbances in FAM^33^, and Zn has an antagonistic relationship in this phenomenon^34^. The five FAM related genes involved in Cu analysis take part in the cytoplasmic portion of the pathway, in which fatty acids biosynthesis occurs by the addition of one or more acetyl-CoA molecules, doubling the number of carbons in the fatty acid molecule produced in each cycle, as per KEGG data (https://www.genome.jp/kegg-bin/show_pathway?map01212).

Fatty acid biosynthesis can start with the co-enzyme Acetyl-CoA carboxylase, the product of *ACACA*, that catalyzes the carboxylation of acetyl-CoA to malonyl-CoA^35^. Subsequently, the product of *FASN* is responsible for the elongation of the fatty acid chains to precursors with 16 carbons. The elongation to 18 carbons requires the product of *ELOVL6*^35^. After that, the Stearoyl-CoA desaturase enzyme, which is the product of *SCD*, catalyzes the synthesis of Oleic acid^31^. Cu is a cofactor of this enzyme^36^ and, in the presence of this mineral, the FAM progresses just until the production of fatty acids with 20 carbons by the product of *ELOVL5*^31^, because it inhibits the production of Linoleic acid by increasing the Oleic acid synthesis^36^. The downregulation of *ACACA*, *FASN*, *ELOVL6*, *SCD*, and *ELOVL5* in the H-Cu group can explain the inhibition of long-chain fatty acids and fat accumulation under low Cu.

A second hypothesis is that malonyl-CoA can also be the switch from fatty acids biosynthesis to fatty acids oxidation and energy production, which can lead to less fatty acid biosynthesis^35^. In rabbits, copper supplementation in the diet decreased the intramuscular fat content by improving fatty acid uptake and fatty acid oxidation^37^. This switch depends on the regulation of malonyl-CoA. For example, in ketosis, ketonic bodies accumulate in the tissue, and the activation of malonyl-CoA activates AMPK. This activation breaks malonyl-CoA, stopping the biosynthesis and starting the oxidation of fatty acids^35^. The AMPK signaling pathway was enriched for Cu.

The second hypothesis can be reinforced by the simultaneous presence among DEGs for Cu of the genes *FASN*, *ACACA* and *SCD*, belonging both to AMPK and FAM pathways, as well as *ADIPOQ*, *PCK2* and *LEP* genes, which are exclusive from the AMPK pathway. Thus, animals with less Cu can have higher fat accumulation by biosynthesis (FAM)^38^ or oxidation (AMPK signaling pathway)^37^; probably by both processes.

Zn is a known Cu antagonist in FAM, due to its role in the stimulation of linoleic acid desaturation^38^. In the Zn analysis, we did not identify the *ACACA* gene as a DEG. Therefore, we hypothesized that, in this case, the product of *FASN* does the first step of fatty acid synthesis. As already discussed, the pathway continues to the precursor of oleic acid. However, in the presence of high Zn, the pathway does not stop on fatty acids with 20 carbons^29^. Zn stimulates the linoleic acid desaturation and the production of long-chain fatty acids^29^.

In Japanese Black Cattle, there is a low negative correlation between Cu concentration and oleic acid (−0.15), between Cu and linoleic acid (−0, 29), and between Zn and linoleic acid (−0.05)^39^. This breed has more intramuscular fat than European cattle breeds. In our population, we did not identify a significant correlation between the GEBVs for oleic acid and the GEBVs for Zn and Cu concentration. We found a weak positive correlation (r = 0.23) between linoleic acid and Cu GEBVs (data not shown). The absence of higher correlations can be attributed to the little variation of these minerals^15^ and fat deposition in our samples^40^. The samples used in the two contrasting groups for Cu and Zn analyses did not present significant (p > 0.05) difference for seven fatty acids concentrations obtained elsewhere^41^ (data not shown). Also, our animals did not exhibit a clinical deficiency of these minerals. Thus, we can assume that, even if the difference in expression did not lead to a significant increase in fat, animals with low Cu concentration present modifications in FAM.

PPAR signaling pathway, enriched in Cu analysis, was also identified and is related to FAM. PPAR is one of the significant adipogenesis activators^42^. Only *PLIN1* gene was in the other fatty acid associated pathways. This gene was found downregulated in Cu, like all the other FAM related genes, and its product is involved directly in lipid metabolism^43^ and adipocyte differentiation^44^. *LEP* gene is also related to Cu and Zn and has an alleged role in the PPAR pathway regulation. It has a well-known relationship with obesity and stimulus for fatty acid oxidation^45^. As all the DEGs mentioned in FAM, *LEP* was upregulated in Zn and downregulated in Cu analyses and interacted with all DEG products in this pathway, when considering Cu, and with *FASN* and *SCD*, when considering Zn. FAM genes were already shown to be related to iron concentration in a differential expression analysis with samples from the same population used in this study^14^.

We retrieved high similarity with known proteins for two non-annotated DEGs for Cu. One of them, downregulated for Cu, is similar to the mouse *SENP3* gene. This gene has high similarity to other SENP family protein gene, *SENP2*^46^. Both encode proteases that release SUMO3 and SUMO2 monomers, involved in several biological processes^46^. Regarding fat deposition, overexpression of *SENP2* increases fatty acid oxidation by upregulating the expression of enzymes linked to this process^47^. This non-annotated DEG can corroborate the hypothesis of the involvement of Cu concentration in fatty acid oxidation in cattle.

The *THRSP* gene, identified as upregulated for Cu and Zn, encodes a nuclear protein involved in fatty acid synthesis^48^ interacting with *FASN* and *ELOVL6*. *THRSP* upregulation activates *FASN*^49^, being a candidate to the mechanism of FAM regulation by Zn.

Among the other four genes downregulated for Cu, *PCK2* is a candidate for obesity^50^ and is part of AMPK and PPAR signaling pathways. This gene’s product interacts with *ACACA*. It has an impact in FAM by receptor interaction and changes in *RBP4* gene, which plays a role in non-alcoholic fatty liver disease and can contribute to insulin resistance^51^. All these genes and pathways linking Cu and Zn to lipid metabolism can explain the genetic mechanisms underlying Cu associations to FAM and Zn antagonism in these processes.

### Pathways enriched for just one mineral

*COMP*, *FCGR3A*, *BLA-DQB*, and *THBS4* genes are involved in the phagosome pathway, all downregulated for K. *BLA-DQB* encodes an antigen, and the other genes encode glycoproteins with already known roles in phagocytosis. Potassium channels are known to modulate changes in the membrane during phagocytosis^52^, which can explain the relationship between the expression of these genes and K concentration.

The genes *C1QB*, *C1QC*, *C7*, and *NCAM1* were downregulated for Ca and partake in the prion disease pathway. The first two genes showed an interaction, and they encode proteins that form the complement component 1, involved in the immune complement system. These genes are linked to the *LAPTM5* gene, which encodes a lysosomal transmembrane protein. *C7* gene encodes a serum protein involved in the immune system and is connected to *C1QTNF3*, a gene that encodes another protein involved in the immune complement system. *NCAM1* gene encodes a protein that is a cell adhesion linked to *CD44*, part of the ECM-receptor pathway, showing that all pathways detected in this study are linked.

## Conclusion

By comparing the expression of genes in muscle samples with contrasting mineral concentrations, we hypothesized that the genetic regulation core for all minerals studied, except Zn, resides in events of extracellular matrix interaction. ECM-receptor interaction, focal adhesion, and PI3k-Akt signaling pathways seem to be related to K, P, Na, Mg, Cu, and Ca content profiles in skeletal muscle. We also pointed out genes that may explain Cu and Zn association to adipogenesis-related pathways, as well as their antagonism on fat accumulation. Future studies can target our raised hypotheses and validate our DEGs to elucidate these biological mechanisms, since our main goal was *in silico* prediction.

## Methods

### Animals

All animal and experimental procedures were carried out following the guidelines provided by the Institutional Animal Care and Use Committee Guidelines of Embrapa Pecuária Sudeste ethics committee (São Carlos, São Paulo, Brazil. Protocol CEUA 01/2013). The Ethical Committee of Embrapa Pecuária Sudeste (São Carlos, São Paulo, Brazil) approved all experimental and animal protocols (approval code CEUA 01/2013). A group of 133 Nelore steers composes our samples that previous projects already used to produce data for mineral concentration^15^, and RNA-Seq^14^. The entire sample group comes from a population of 373 Nelore steers fathered by 34 purebred Nelore sires in half-sibling families.

The animals used in our work result from artificial insemination, were born in two different breeding seasons, in two different farms. Approximately at 21 months of age, all animals used in this research were transferred and maintained in a feedlot at Embrapa Pecuária Sudeste (São Carlos, São Paulo, Brazil). After a 28 days adaptation period, they received food, water, and had a similar nutritional and sanitary management until the slaughter. The animals had *ad libitum* feed access twice a day with 5% refusals, discarded daily. The diet contained 40% of dry matter constituted by corn silage, crude protein, ground corn, soybean meal, cottonseed, soybean hull, limestone, mineral mixture, urea and monensin (Rumensin®).

### Mineral concentration genetic breeding value and contrasting groups

Mineral concentrations were measured as described elsewhere^6^ from *Longissimus thoracis* muscle steaks sampled between 11^th^ and 13^th^ ribs. Briefly, the samples were lyophilized and digested with microwave assistance using a closed-vessel microwave digestion system (Ethos-1600, Milestone-MLS, Sorisole, Italy). The mineral quantification was obtained in the Vista Pro-CCD ICP-OES spectrometer with a radial view (Varian, Mulgrave, Australia). Among measured minerals we selected Calcium (Ca), Copper (Cu), Potassium (K), Magnesium (Mg), Sodium (Na), Phosphorus (P), Sulfur (S), Selenium (Se), and Zinc (Zn) for our analyses because they have distinguished extreme animal groups.

The genetic breeding values (GEBV) for all mineral’s concentration were estimated elsewhere^15^ for 373 animals encompassing our samples using a Bayesian model implemented in GenSel software^53^. The model considered contemporary groups formed by birthplace, feedlot location, and breeding season as fixed effects and age at slaughter as a covariate. The GEBVs were used to select 12 animals for each mineral with extreme phenotypes (six with high GEBV, called H, and six with low GEBV, called L).

### RNA-Seq data

We used muscle samples from all animals in each contrasting group for RNA extraction and RNA-Seq analysis as described elsewhere^14^. Total RNA was extracted using TRIzol^®^ (Life Technologies, Carlsbad, CA). Its integrity was analyzed in a Bioanalyzer 2100^®^ (Agilent, Santa Clara, CA, USA). Library preparation for RNA-Seq analysis was carried out using the TruSeq RNA Sample Preparation Kit (Illumina, San Diego, CA). Sequencing was carried out in an Illumina HiSeq 2500^®^. All laboratory procedures were carried out in ESALQ Genomics Center (Piracicaba, SP, Brazil).

### DEGs identification

RNA-Seq data obtained from muscle samples belonging to contrasting groups for a given mineral were used to determine DEGs for each mineral. The pipeline was as described in^14^ with the insertion of StringTie v1.2.2^54^ instead of Cufflinks^18^ in Tuxedo Suite pipeline^18^.

SeqClean software (http://sourceforge.net/projects/seqclean/files/) was used to trim low-quality sequences and adapters. TopHat software v2.0.11^18^ was used to align reads to the reference bovine genome (*Bos taurus* UMD 3.1, http://www.ensembl.org/Bos_taurus/Info/Index). After that, the StringTie v1.2.2^54^ was used to assemble the transcripts and to estimate their expression levels, normalized as FPKM (fragments per kilobases per million). Cuffdiff v2.2.1^18^ was then used to test for differential expression, calculating the average of each gene expression among the samples of the same contrasting group and calculating the FC. Only transcripts that passed the threshold of at least ten fragments aligned entered the differential expression test.

We performed a functional annotation analysis using Trinotate pipeline (http://trinotate.sourceforge.net/) to identify possible functions of non-annotated and predicted differentially expressed proteins for the minerals.

### Relationship among minerals

We used a pairwise Pearson correlation analysis for the GEBVs of all minerals to quantify their dependency. Also, we performed a Pearson correlation analysis between GEBV and raw concentration measure for each mineral in order to convey the reliability of the GEBVs. A t-test was applied to verify if the mean GEBVs of the samples for all contrasting groups would also be statistically different for any other mineral.

### Biological processes and pathways

We performed enrichment analysis using DAVID v6.8 software^20^ to discover biological processes in which the DEGs are acting. To access known protein-protein interaction regarding DEGs and pathways in which they may participate, we used STRING v10.5 software^55^.

## Supplementary information

Supplementary information
