# Supplementary material for: Muscle transcriptome analysis reveals genes and metabolic pathways related to mineral concentration in Bos indicus
Source: Sci Rep. 2019 Sep 3;9:12715. doi: 10.1038/s41598-019-49089-x (PMC6722098; doi:10.1038/s41598-019-49089-x)

Muscle transcriptome analysis reveals genes and metabolic pathways related to mineral concentration in *Bos indicus*

Juliana Afonso[1], Luiz Lehmann Coutinho[2], Polyana Cristine Tizioto[2], Wellison Jarles da Silva Diniz[1], Andressa Oliveira de Lima[1], Marina Ibelli Pereira Rocha[1], Carlos Eduardo Buss[1], Bruno Gabriel Nascimento Andrade[3], Otávio Pyaia[1], Juliana Virginio da Silva[4], Laura Albuquerque Lins[5], Caio Fernando Gromboni[6], Ana Rita Araújo Nogueira[3], Marina Rufino Salinas Fortes[7], Gerson Barreto Mourão[2], Luciana Correia de Almeida Regitano[*3]

[1]Department of Evolutionary Genetics and Molecular Biology, Federal University of São Carlos, São Carlos, Brazil, [2]Department of Animal Science, University of São Paulo/ESALQ, Piracicaba, Brazil, [3]Embrapa Pecuária Sudeste, São Carlos, Brazil,[4]Physics Institute of São Carlos, University of São Paulo, São Carlos, Brazil, [5]Animal Science department, Laboratory of Molecular Genetics. São Paulo State University, Jaboticabal, Brazil, [6]Bahia Federal Institute of Education, Science and Technology, Ilhéus, Brazil, [7]School of Chemistry and Molecular Biosciences, Faculty of Sciences, The University of Queensland, Brisbane, Australia. [*]Corresponding author.
**Correspondence to: luciana.regitano@embrapa.br*

Supplementary Table S1. Log2fold_change for each DEG in each mineral analysis. Genes with positive log2fold_change are upregulated in the high mineral groups in relation to the low mineral groups. Genes with negative log2fold_change are downregulated in the high mineral groups in relation to the low mineral groups.

Gene	Ca	Cu	P	Mg	Se	Zn	K	S	Na
ADAM12	-2.33876	-2.42863	-1.56725	-1.61797	-1.96537	-	-1.6051	-	-
ADAMTS12	-0.89906	-1.08832	-	-	-	-	-	-	-
ADAMTS2	-0.71449	-0.69446	-	-	-	-	-	-	-
AEBP1	-1.19137	-1.65381	-1.24569	-1.26387	-	-	-1.26926	-	-1.49998
AIF1L	-0.71217	-	-	-	-	-	-	-	-
AMOTL2	-0.71154	-	-	-	-	-	-	-	-
ANGPTL2	-0.81685	-	-	-0.776	-	-	-0.86545	-	-
ANTXR2	-0.73518	-	-	-	-	-	-	-	-
APOO	0.684682	-	-	-	-	-	-	-	-
ARSA	-1.01411	-	-	-	-	-	-	-	-
BASP1	-1.20745	-	-	-	-	-	-	-	-
BGN	-0.87363	-	-	-	-	-	-	-	-
BLA-DQB	-1.24945	-	-	-1.04454	-	-	-0.84441	-	-0.86674
C1QB	-0.85895	-0.78118	-	-	-	-	-	-	-
C1QC	-0.8805	-0.8562	-	-	-	-	-	-	-
C1QTNF3	-1.41821	-	-	-	-	-	-	-0.86194	-
C1QTNF6	-1.44409	-1.66404	-1.15404	-1.15348	-	-	-1.15973	-	-1.19421
C4H7orf41	-0.75216	-	-	-	-	-	-	-	-
C7	-0.85588	-	-	-	-	-	-	-	-
CCDC3	-1.50628	-1.67875	-	-	-	-	-	-	-1.15013
CCDC80	-0.82888	-	-	-	-	-	-	-	-
CD44	-0.77317	-0.99736	-0.89846	-0.93892	-	-	-0.92045	-	-0.89702
CDH11	-0.78841	-	-	-	-	-	-	-	-
CDON	-0.9255	-0.94615	-	-	-	-	-	-	-
CHODL	-1.05637	-	-	-	-	-	-	-	-
CIDEC	-1.39124	-1.59464	-	-	-	-	-	-	-
CILP2	-2.39317	-3.74297	-2.78695	-2.72521	-3.0268	-	-2.78263	-	-

CKAP4	-0.75602	-	-	-	-	-	-	-	-
CLPTM1L	-0.65322	-	-	-	-	-	-	-	-
CNN1	0.711888	-	-	-	-	-	-	-	-
COL11A1	-2.38526	-3.7989	-2.18555	-2.22474	-3.74735	-	-2.04408	-2.14354	-2.29603
COL11A2	-1.14903	-1.3406	-1.83547	-1.41365	-	-	-1.86117	-	-1.59795
COL12A1	-1.49819	-2.09503	-	-1.21789	-1.77317	-	-	-	-1.34935
COL13A1	-1.8361	-	-	-	-	-	-	-	-
COL21A1	-1.24837	-	-	-	-	-	-	-	-
COL22A1	-1.59205	-3.1081	-1.95923	-1.87233	-2.41876	-	-1.81946	-	-2.15377
COL5A1	-0.88132	-	-	-	-	-	-	-	-
COL5A2	-1.02558	-1.04413	-	-	-	-	-	-	-
COMP	-2.78746	-4.74229	-2.39792	-2.44529	-3.88935	-	-2.42829	-1.99361	-2.58822
COX7A1	0.669829	-	-	-	-	-	-	-	-
CPXM2	-1.59315	-2.06927	-1.56937	-1.5262	-1.9932	-	-1.53876	-	-1.55684
CREB3L2	-0.85959	-	-	-	-	-	-	-	-
CTHRC1	-1.40659	-1.59226	-1.41587	-	-	-	-1.40371	-	-
CYCS	0.681419	-	-	-	-	-	-	-	-
DDR2	-0.68622	-	-	-	-	-	-	-	-
DKK2	-0.91717	-	-	-	-	-	-	-	-
DPY19L1	-0.80382	-	-	-	-	-	-	-	-
DPYSL2	-0.68176	-	-	-	-	-	-	-	-
ELOVL5	-0.94836	-1.20225	-	-	-	-	-	-	-
ELOVL6	-1.68163	-2.31682	-	-	-	1.55465	-	-1.58213	-
EMILIN1	-0.78729	-	-	-	-	-	-	-	-
F13A1	-0.86547	-1.00129	-	-	-	-	-	-	-
FAM105A	-0.94664	-	-	-	-	-	-	-	-
FAM129A	-0.71816	-0.73375	-	-	-	-	-	-	-
FAM198B	-0.68533	-	-	-	-	-	-	-	-
FBLN7	-2.10962	-2.95252	-2.18325	-2.02096	-2.57333	-	-2.18056	-	-2.22076
FMOD	-1.34096	-1.73548	-	-	-1.53591	-	-	-	-1.06552

FREM1	-1.4577	-	-	-1.33748	-	-	-1.34987	-	-1.35464
FZD4	-0.88642	-	-	-	-	-	-	-	-
GALNTL1	-0.88913	-	-	-	-	-	-	-	-
GAS7	-0.95636	-	-	-	-	-	-	-	-
GATM	-0.89744	-	-	-	-	-	-	-	-
GBP4	1.99192	-	-	-	-	-	-	-	-
G0I1	-1.17206	-1.36475	-	-	-	-	-	-	-
GPC6	-0.97321	-1.03403	-	-	-	-	-	-	-
HIST1H2AC	1.00191	-	0.904241	1.08215	-1.07653	0.964853	-	1.18544	0.996791
HIST1H2BD	0.765682	-	-	-	-	-	-	-	-
HOXA9.MIR196B	-4.10845	-	-	-	-	-	-	-	-
HSPH1	0.787265	-	-	-	-	-	-	-	-
IFI27	1.51807	-	-	-	-	-	-	-	1.47733
IFI44	1.66291	-	-	-	-	-	-	-	-
IGF2	-0.75924	-	-	-0.83875	-	-	-	-	-
IGFBP4	-0.8346	-	-	-	-	-	-	-	-
IGSF3	-0.85875	-	-	-	-	-	-	-	-
ISLR	-0.80689	-	-	-	-	-	-	-	-
ITGA11	-0.94251	-	-	-	-	-	-	-	-
ITGBL1	-0.85322	-	-	-	-	-	-	-	-
ITIH5	-0.80458	-	-	-	-	-	-	-	-
KCNK2	-2.70444	-2.28758	-	-	-	-	-	-	-
KERA	-1.29835	-	-	-	-	-	-	-	-
KY	-0.98094	-	-	-	-	-	-	-	-
LAPTM5	-0.67845	-	-	-	-	-	-	-	-
LASP1	-0.71898	-	-	-	-	-	-	-	-
LEP	-1.40113	-1.95029	-	-	-	1.54989	-	-	-
LEPREL1	-1.0899	-	-1.45191	-1.4066	-	-	-1.41545	-	-1.51631
LMF1	-1.0081	-	-	-	-	-	-	-	-
LOC100138864	0.907404	-	-	-	-	-	-	-	-

LOC100335754	-0.9179	-	-	-1.04611	-	-0.944	-	-	-
LOC100336629	-0.73639	-	-	-	-	-	-	-	-
LOC100336823	0.785098	-	-	-	-	-	-	-	-
LOC100337023	-1.04622	-	-	-	-	-	-	-	-
LOC100337426	1.4257	-	-	-	-	-	-	-	-
LOC100847340	1.03953	-	-	-	-	-	-	-	-
LOC100847413	0.980216	-	-	-	-	-	-	-	-
LOC100848095	-1.12607	-	-	-	-	-	-	-	-
LOC100848852.LOC784007	-1.00651	-	-	-	-	-	-	-	-
LOC100848883	1.60741	-	-	-	-	-	-	-	-
LOC100848913	-1.38656	-	-	-	-	-	-	-	-
LOC508347	1.12422	-	-	-	-	-	-	-	-
LOC520070	-0.98493	-	-	-	-	-1.05236	-	-	-
LOC535166	-0.84796	-0.90184	-	-	-	-	-	-	-
LOC615589	-0.68678	-	-	-	-	-	-	-	-
LOC618422	1.85792	1.54226	-	-	1.52097	-	-	-	-
LOC781339	0.732983	-	-	-	-	-	-	-	-
LOC784243	-2.4827	-	-	-	-	-	-	-	-
LOC785386	-2.78241	-	-	-	-	-	-	-	-
LOC786652	-0.70417	-	-	-	-	-	-	-	-
LOC786948	-1.20883	-	-	-	-	-1.29715	-	-	-
LOC787269	1.43183	-1.58242	-	-	-1.78462	-	-	-	-
LOC787803	3.46185	-2.88534	-	-	-	-	-	-	-
LOX	-1.15943	-1.11357	-	-	-	-	-	-	-
LOXL2	-0.7307	-0.96013	-	-	-	-	-	-	-
LPL	-0.89189	-	-	-	-	-	-	-	-
LTBP2	-1.16011	-1.36893	-	-	-	-	-	-	-
LUM	-1.01106	-0.6896	-	-	-	-	-	-	-
MAFB	-0.9991	-	-	-	-	-	-	-	-
MAN1A1	-0.75205	-	-	-	-	-	-	-	-

MARCKS	-1.2165	-	-	-	-	-	-	-	-
MDFIC	-0.69597	-	-	-	-	-	-	-	-
MEST	-1.74506	-1.20056	-1.54635	-1.55164	-	-	-1.53333	-	-
MGC148714	0.802315	-	-	-	-	-	-	-	-
MGST1	-0.95802	-1.06255	-	-	-	-	-	-	-
MKX	-2.62921	-2.16954	-	-	-	-	-	-	-
MMP14	-1.03737	-1.04033	-	-	-	-	-	-	-
MMP16	-1.30873	-	-	-0.99617	-	-	-	-	-
MMP2	-1.09849	-1.00009	-	-	-	-	-	-	-
MPEG1	-0.98128	-0.85838	-	-0.85201	-	-	-	-	-0.89755
MPZ	-1.0218	1.06504	-	-	-	-0.97768	-1.01442	-	-
MRC2	-0.80892	-	-	-	-	-	-	-	-
MRPL48	0.717858	-	-	-	-	-	-	-	-
MX1	1.11258	-	-	-	-	1.2064	-	-	-
MXRA5	-1.96682	-2.29902	-1.58276	-1.55165	-1.46552	-	-1.59087	-	-1.75447
MYADM	-0.7596	-	-	-	-	-	-	-	-
NCAM1	-0.79383	-	-1.09067	-0.96821	-	-	-1.0385	-	-
NDUFA12	0.687429	-	-	-	-	-	-	-	-
NDUFA4	0.792351	-	-	-	-	-	-	-	-
NFE2L3	0.890982	-	-	-	-	-	-	-	-
NID2	-0.69875	-	-	-	-	-	-	-	-
NOTCH2	-0.78415	-	-	-	-	-	-	-	-
NTRK2	-0.84958	-	-	-	-	-	-	-	-
OAF	-0.94771	-	-	-	-	-	-	-	-
OAS1.OAS2	1.12245	-	-	-	-	-	-	-	-
ODZ3	-1.49037	-1.45245	-1.35792	-1.32339	-	-	-1.37956	-	-1.58755
OLFML2A	-0.7353	-	-	-	-	-	-	-	-
OLFML2B	-1.05655	-0.89426	-0.87362	-0.89391	-	-	-0.87554	-	-0.98673
OLFML3	-0.69668	-	-	-	-	-	-	-	-
PCDH18	-0.91973	-	-	-	-	-	-	-	-

PCK2	-1.66627	-1.73633	-	-	-	-	-	-	-
PCOLCE	-0.74991	-	-	-	-	-	-	-	-
PDGFD	-0.86213	-	-	-	-	-	-	-	-
PDGFRA	-0.63329	-	-	-	-	-	-	-	-
PEG10	-1.199	-	-	-	-	-	-	-	-
PI16	-0.72356	-	-	-	-	-	-	-	-
PLEKHA5	-0.92384	-1.31875	-	-	-	-	-	-	-
PLIN1	-1.23407	-1.20362	-	-	-	-	-	-	-
PLXDC1	-0.78505	-	-	-	-	-	-	-	-
POSTN	-1.50338	-2.57708	-1.53251	-1.59063	-	-	-1.51442	-	-1.59107
PPP1R1B	-1.05471	-	-	-	-	-	-	-	-
PRELP	-0.88235	-	-	-	-	-	-	-	-
PRRX2	-1.30625	-1.30948	-1.36988	-1.31027	-	-	-1.44173	-	-1.45194
PTGFRN	-0.8317	-	-	-	-	-	-	-	-
PTMS	-0.65293	-	-	-	-	-	-	-	-
QSOX1	-0.65475	-	-	-	-	-	-	-	-
RBP4	-0.85378	-1.25747	-	-	-	-	-	-	-
RCAN1	1.29609	-	-	-	-	-	-	-	-
RGS2	1.58863	-	1.27246	1.65305	-	-	1.30084	-	1.23359
SCARA3	-1.07318	-1.20056	-	-	-	-	-	-	-
SCD	-1.29814	-1.26175	-	-	-	0.986182	-	-	-
SCIN	-0.88724	-	-	-	-	-	-	-	-
SCN3B	0.912329	-	-	1.18107	-	-	1.13383	-	-
SDC3	-0.84636	-	-	-	-	-	-	-	-
SELRC1	0.8018	-	-	-	-	-	-	-	-
SERPINF1	-0.65572	-	-	-	-	-	-	-	-
SESN3	-0.96268	-0.81352	-	-	-	-	-	-	-
SFRP4	-1.11038	-	-	-	-	-	-	-	-
SFRP5	-1.21423	-	-	-	-	-	-	-	-
SH3BGRL3	-0.79043	-	-	-	-	-	-	-	-

SLC16A2	-1.01542	-	-	-	-	-	-	-	-
SLCO1A2	-0.84934	-	-	-	-	-	-	-	-
SMPDL3A	-0.96603	-	-	-	-	-	-	-	-
SPON2	-1.07332	-	-	-	-	-	-	-	-
SPRY4	-0.84754	-	-	-	-	-	-	-	-
SYT4	-5	-	-	-	-	-	-	-	-
TF	-2.09013	-	-	-	-	-	-	-	-
THRSP	-1.85297	-2.73927	-	-	-	1.69724	-	-1.77612	-
THY1	-0.82247	-	-	-	-	-	-	-	-
TIMP2	-0.74105	-	-	-	-	-	-	-	-
TMEM119	-1.8185	-1.73674	-	-	-	-	-	-	-
TMEM233	0.714143	-	-	-	-	-	-	-	-
TNMD	-2.34372	-4.76042	-2.87619	-2.9694	-4.04006	-	-2.77153	-2.35386	-2.90605
TRIL	-1.36045	-	-	-	-	-	-	-	-
TUSC5	-1.57975	-1.5676	-	-	-	-	-	-	-
USMG5	1.03367	-	-	-	-	-	-	-	-
WIPF1	-0.8782	-	-	-	-	-	-	-	-
ACACA	-	-1.05437	-	-	-	-	-	-	-
ACSM1	-	-1.31376	-0.92468	-	-	-	-	-	-1.22592
ACTC1	-	-1.40947	-2.49413	-1.31724	-	-	-2.49055	-	-
ADIPOQ	-	-1.06379	-	-	-	-	-	-	-0.94059
AGTPBP1	-	0.955303	-	-	-	-	-	-	-
ANXA1	-	-0.97355	-	-	-	-	-	-	-
ANXA2	-	-0.83481	-	-	-	-	-	-	-
BHLHE40	-	-0.76351	-	-	-	-	-	-	-
C14H8orf22	-	0.863786	-	-	-	-	-	-	-
C28H10orf116	-	-0.96883	-	-	-	-	-	-	-
CD109	-	-0.74126	-	-	-	-	-	-	-
CD163	-	-0.78423	-	-	-	-	-	-	-
CHI3L1	-	-1.03364	-	-	-	-	-	-	-

CHPF	-	-0.81947	-	-	-	-	-	-	-
COL18A1	-	-0.76903	-0.75774	-	-	-	-	-	-0.85112
CPM	-	-1.34574	-	-0.96001	-	-	-	-	-1.06703
DAB2	-	-0.82736	-	-	-	-	-	-	-
DAP	-	-0.7027	-	-	-	-	-	-	-
DGAT2	-	-1.0234	-	-	-	-	-	-	-
D0JB1	-	0.789664	-	-	-	-	-	-	-
EBF1	-	-0.73079	-	-	-	-	-	-	-
ELMO1	-	-0.91782	-	-	-	-	-	-	-
EMP1	-	-0.86392	-	-	-	-	-	-	-
FASN	-	-1.87604	-	-	-1.16049	0.950755	-	-	-
FAT1	-	-0.96943	-	-	-	-	-	-	-
FNDC3B	-	-0.73871	-	-	-	-	-	-	-
GAS2	-	-2.89816	-	-	-	-	-	-	-
GLCE	-	-0.73931	-	-	-	-	-	-	-
GSTM3	-	1.0793	-	-	-	-	-	-	-
HSPA6	-	2.64101	-1.24581	-	-	-1.68298	-	2.32092	-
ITGA10	-	-1.55666	-1.44803	-1.53707	-	-	-1.41923	-	-1.68951
KRT8	-	1.73887	-	-	-	-1.45334	-	-	-
LOC100300267	-	-1	-	-	-	-	-	-	-
LOC100337216.LOC520016	-	1.51682	-1.42212	-1.32589	-	-	-1.55129	-	-
LOC100337244	-	2.3195	-	-	-	-	-	-	-
MAL2	-	-1.31285	-	-	-	-	-	-	-
MLLT11	-	-2.2348	-	-	-	-	-	-	-
MT1A	-	-1.73224	-	-	-	-	-	-	-
MT2A	-	-1.1827	1.77149	2.0783	-	1.26086	1.89059	1.21384	1.89389
MYH10	-	-0.80772	-	-	-0.77608	-	-	-	-
NECAB3	-	1.22668	-	-	-	-	-	-	-
NOV	-	-0.90966	-	-	-	-	-0.73819	-	-0.75658
P4HA3	-	-1.07926	-	-	-	-	-	-	-

PDE3B	-	-1.24654	-	-	-	-	-	-	-
PDZD2	-	-0.8555	-	-	-	-	-	-	-
PERP	-	-1.90158	-2.49873	-2.18506	-2.62239	-	-2.42679	-	-1.9586
PI15	-	-1.8518	-	-	-	-	-	-	-2.32329
PMP2	-	2.48424	-	-	-	-	-	-	-
PON3	-	2.24936	-	-	2.83053	1.94258	-	-	-
PPL	-	-0.85512	-	-	-	-	-	-	-
PTGIR	-	-2.51609	-	-	-	-	-	-	-
PYCR1	-	-1.39843	-	-	-	-	-	-	-
RCN3	-	-1.00603	-	-	-	-	-0.81136	-	-
RET	-	-0.76962	-	-	-	-	-	-	-
S100A10	-	-0.88141	-	-	-	-	-	-	-
SFXN1	-	-1.31849	-	-	-	-	-	-	-
SLC6A4	-	-1.39085	-	-	-	-	-	-	-
SPP1	-	-1.33087	-	-	-	-	-	-	-
SRXN1	-	-1.37719	-0.90198	-	-	-	-	-	-
SYT11	-	-1.2163	-	-	-	-	-	-	-
THBS1	-	-1.41829	-	-	-	-	-	-	-
THBS4	-	-2.81465	-1.90816	-1.97004	-	-	-1.91339	-	-2.14173
TKT	-	-0.86569	-	-	-	-	-	-	-
TNC	-	-2.20309	-1.99968	-1.97766	-1.52174	-	-2.02989	-	-2.15934
TNFRSF12A	-	-0.87425	-	-	-	-	-	-	-
TPBG	-	-1.66784	-1.80966	-1.67355	-	-	-1.77094	-	-1.48611
TRAFD1	-	-0.73233	-	-	-	-	-	-	-
UCK2	-	-1.08919	-	-	-1.27875	-	-	-	-
VCAN	-	-0.75408	-	-	-	-	-	-	-
WISP1	-	-2.68722	-	-	-	-	-	-	-
CES1	-	-	-1.03758	-1.01539	-	-	-0.9397	-	-
CRABP2	-	-	-2.30287	-2.14704	-	-	-2.33593	-	-1.72625
CRTAC1	-	-	-2.05002	-2.04274	-	-	-2.06918	-	-

CYP4B1	-	-	-1.4632	-	1.66771	-	-1.11333	-	-
FCGR3A	-	-	-1.25109	-	-	-	-1.31582	-	-1.28899
HES1	-	-	0.753489	-	-	-	-	-	-
KLF5	-	-	-0.97657	-	-	-	-0.98264	-	-
LOC100847238	-	-	1.11704	1.25662	-0.85139	-	1.23825	-	-
LOC100848544	-	-	1.01269	1.06664	-	-	1.10129	-	-
LOC100848726	-	-	0.840227	-	-	-1.5957	1.03767	2.31839	1.11774
LOC100848920	-	-	0.98718	0.982841	-	-	1.07905	-	-
LOC515150	-	-	-0.89214	-	-	-	-	-	-0.898
NES	-	-	-0.85122	-0.81965	-	-	-0.92603	-	-
S100A4	-	-	-0.86834	-0.91138	-	-	-0.8845	-	-0.84248
APOE	-	-	-	-0.97694	-	-	-0.91763	-	-0.8511
GADD45A	-	-	-	-1.03297	-	-	-	-	-
GADL1	-	-	-	0.730109	-	-	-	-	0.689153
KCNC4	-	-	-	1.03166	-	-	-	-	1.13707
LRRC20	-	-	-	0.756225	-	-	0.689864	-	0.654978
MAOB	-	-	-	-1.10237	-	-	-1.12386	-1.66064	-1.11835
MGP	-	-	-	-0.80258	-	-	-	-	-
SLIT3	-	-	-	-0.85412	-	-	-0.91309	-	-0.91819
TMSB4	-	-	-	-0.8505	-	-	-0.85618	-	-
UCHL1	-	-	-	-0.92775	-	-	-	-	-
ACTA2	-	-	-	-	0.858818	-	-	-	-
AMPD3	-	-	-	-	-0.89207	-	-	-	-
DLK1	-	-	-	-	0.829306	-	-	-	-
ECHDC2	-	-	-	-	1.00543	-	-	-	-
LOC100848346	-	-	-	-	2.60288	-	-	-	-
LOC100848684	-	-	-	-	1.96408	-	-	-	-
LOC789192	-	-	-	-	-1.30828	-	-	-	-
NR4A2	-	-	-	-	0.973619	-	-	-	-
RN5-8S1	-	-	-	-	2.79644	-2.83236	-	2.71751	-

AKAP9	-	-	-	-	-	0.784405	-	-	-
ANGPTL4	-	-	-	-	-	-0.79863	-	-	-
BANK1	-	-	-	-	-	1.3818	-	-	-
C1QTNF1	-	-	-	-	-	-0.87504	-	-	-
CISH	-	-	-	-	-	0.894319	-	0.960376	-
CLDN5	-	-	-	-	-	-0.90859	-	-	-
CYGB	-	-	-	-	-	-0.99274	-	-	-
IFI6	-	-	-	-	-	1.23173	-	-	-
ISG15	-	-	-	-	-	1.74381	-	-	1.5418
MGLL	-	-	-	-	-	-0.96588	-	-	-
PLIN5	-	-	-	-	-	-0.88872	-	-	-
RN28S1	-	-	-	-	-	-1.12057	-	-	0.939374
ROCK2	-	-	-	-	-	0.827486	-	-	-
UCP2	-	-	-	-	-	-1.18504	-	-	-
CREM	-	-	-	-	-	-	0.830859	-	-
DKK3	-	-	-	-	-	-	-1.00825	-	-0.96566
HPCAL4	-	-	-	-	-	-	-	-1.40152	-
LOC100141258	-	-	-	-	-	-	-	-2.81272	-
LOC100337053	-	-	-	-	-	-	-	-1.68767	-
MYLK3	-	-	-	-	-	-	-	-1.89817	-
SPOCK2	-	-	-	-	-	-	-	-1.13519	-
KCNMB4	-	-	-	-	-	-	-	-	-0.89854
LOC100299180	-	-	-	-	-	-	-	-	-1.57076
LOC100336936	-	-	-	-	-	-	-	-	-1.58976
LOC100847553	-	-	-	-	-	-	-	-	1.03644
LYZ	-	-	-	-	-	-	-	-	-1.16529
NDUFS6	-	-	-	-	-	-	-	-	0.76202
PRDM8	-	-	-	-	-	-	-	-	-1.38748
SMTNL1	-	-	-	-	-	-	-	-	0.783364
TPSB1	-	-	-	-	-	-	-	-	-1.15721

Supplementary Table S2. Significant Trynotate annotation results for the non-annotated DEGs.

Top BLASTX hit	Top BLASTP hit	eggnog	Kegg	Mineral
Myoregulin {ECO:0000303|PubMed:25640239}	.	.	KEGG:hsa:100507027	Cu
Sentrin-specific protease 3	.	COG5160 SUMO1 sentrin specific peptidase	KEGG:mmu:80886`KO:K08593	Cu
Putative deoxyribonuclease TATDN1	.	COG0084 tatd family	KEGG:bta:509365`KO:K03424	Cu
Sentrin-specific protease 3	.	COG5160 SUMO1 sentrin specific peptidase	KEGG:mmu:80886`KO:K08593	Cu
Ig gamma-3 chain C region	Ig gamma-3 chain C region	.	.	P
LINE-1 retrotransposable element ORF2 protein	.	ENOG410Y9TZ NA	.	P
.	Endogenous retrovirus group V member 2 Env polyprotein	ENOG4111C12 endogenous retrovirus group MER34	KEGG:hsa:100271846	P
RNA-directed DNA polymerase from mobile element jockey	.	.	.	P
RNA-binding protein 39	RNA-binding protein 39	ENOG410XP20 RNA binding motif protein	KEGG:pon:100172241`KO:K13091	P
LINE-1 retrotransposable element ORF2 protein	.	ENOG410Y9TZ NA	.	Mg
.	Endogenous retrovirus group V member 2 Env polyprotein	ENOG4111C12 endogenous retrovirus group MER34	KEGG:hsa:100271846	Mg
Retrovirus-related Pol polyprotein from type-1 retrotransposable element R2	.	.	.	Mg
RNA-directed DNA polymerase from mobile element jockey	.	.	.	Mg
Pol polyprotein	.	.	KEGG:vg:22318531	Mg

Deoxynucleotidyltransferase terminal-interacting protein	Deoxynucleotidyltransferase terminal-interacting protein 1			Mg
Ig gamma-3 chain C region	Ig gamma-3 chain C region	.	.	Mg
LINE-1 retrotransposable	.	ENOG410Y9TZ NA	.	K
.	Endogenous retrovirus group V member 2 Env polyprotein	ENOG4111C12 endogenous retrovirus group MER34	KEGG:hsa:100271846	K
Retrovirus-related Pol polyprotein	.	.	.	K
RNA-directed DNA polymerase	.	.	.	K
Engulfment and cell motility protein 2	Engulfment and cell motility protein 2	ENOG410XPKN Engulfment and cell motility	KEGG:bta:508361`KO:K18985	K
L-lactate dehydrogenase A chain	.	.	.	Na
RNA-directed DNA polymerase	.	.	.	Na
Kinesin-like protein KIF3B	Kinesin-like protein KIF3B	COG5059 Kinesin family member	KEGG:hsa:9371`KO:K20196	Na
Immunoglobulin heavy constant gamma 2 {ECO:0000303|PubMed:11340299	Immunoglobulin heavy constant gamma 2 {ECO:0000303|PubMed:11340299| ECO:0000303|Ref.13}	.	.	Na
.	Putative uncharacterized transposon-derived protein F52C9.6	.	.	Zn

Supplementary Table S3. Significant Pearson correlations among all the GEBVs for all the minerals and associated p-values.

Ca	Cu	Mg	P	K	Se	Na	S	Zn
**Ca**	0.22822	0.61979	0.64251	0.61957		0.58770	0.63537	0.59900
**p-value**	0.0082	<.0001	<.0001	<.0001		<.0001	<.0001	<.0001
**Cu**		0.26372	0.24692	0.24173	0.19672	0.21535	0.20790	
**p-value**		0.0022	0.0042	0.0051	0.0232	0.0128	0.0163	
**Mg**			0.96943	0.97012	-0.20101	0.90066	0.79799	0.77813
**p-value**			<.0001	<.0001	0.0203	<.0001	<.0001	<.0001
**P**				0.96956	-0.28677	0.89588	0.82373	0.79786
**p-value**				<.0001	0.0008	<.0001	<.0001	<.0001
**K**					-0.25147	0.88504	0.81317	0.78853
**p-value**					0.0035	<.0001	<.0001	<.0001
**Se**						-0.23626	-0.21005	-0.23024
**p-value**						0.0062	0.0152	0.0077
**Na**							0.79135	0.76104
**p-value**							<.0001	<.0001
**S**								0.66939
**p-value**								<.0001
**Zn**								
**p-value**								

Supplementary Table S4. Significant Pearson correlations between the GEBVs for each mineral and their respective raw mineral concentration. All p-values are <.0001.

Mineral	Ca	Cu	Mg	P	K	Se	Na	S	Zn
**Correlation**	0.777	0.847	0.860	0.857	0.836	0.849	0.836	0.865	0.848

Supplementary Table S5. Average GEBV for all minerals in each contrasting group and the p-value of the tests of significance (t-test) between the extreme group samples' GEBVs for each mineral inside each contrasting group.[a]FDR correction of the p-value for each t-test. The average GEBVs for the original mineral for each group are in bold.

Ca_samples	Ca	Cu	Mg	P	K	Se	Na	S	Zn	Group
NE3	**0.0956**	0.0392	0.0617	0.0759	0.072	-0.125	0.0905	0.0795	0.0919	high
NE7	**0.138**	0.0125	0.0598	0.0765	0.0813	-0.152	0.0719	0.0815	0.134	high
NE19	**0.108**	0.0283	0.0797	0.0845	0.083	-0.0695	0.0959	0.099	0.0993	high
NE33	**0.11**	0.0533	0.0595	0.0687	0.0704	-0.072	0.0552	0.0751	0.0862	high
NE36	**0.111**	0.0624	-4.00E-04	0.0017	2.00E-04	0.0706	0.0095	0.0351	0.0064	high
NE44	**0.257**	-0.015	0.0279	0.0368	0.0256	-0.0243	0.0114	0.0692	0.0201	high
NE1	**-0.0949**	-0.0584	-0.0231	-0.0318	-0.0163	0.0125	-0.0049	-0.0104	-0.0411	low
NE12	**-0.108**	-0.0637	-0.0513	-0.0513	-0.0523	-0.189	-0.061	-0.0628	-0.0551	low
NE18	**-0.131**	-0.063	-0.0205	-0.0257	-0.02	0.0541	-0.0292	0.0067	-0.0285	low
NE27	**-0.109**	-0.0268	-0.0396	-0.0458	-0.0366	-0.163	-0.0528	-0.0353	-0.037	low
NE40	**-0.135**	-0.0044	-0.0173	-0.0055	-0.0147	-9.00E-04	0.0193	-0.0033	-0.0484	low
NE42	**-0.0955**	-0.0128	-0.0112	-0.0231	-0.0219	0.0116	-0.0066	-0.0311	0.033	low
Corrected p-value[a]	**3.85E-04**	2.39E-03	1.37E-03	1.26E-03	1.99E-03	7.66E-01	3.47E-03	3.49E-04	2.99E-03	

Cu_samples	Ca	**Cu**	Mg	P	K	Se	Na	S	Zn	Group
NE15	0.0084	**0.173**	-0.0139	-0.0269	-0.0206	0.0867	-0.019	-0.0081	-0.0629	high
NE23	-0.0492	**0.291**	-0.0121	-0.0223	-0.0311	0.11	-0.0222	-0.0327	-0.0745	high
NE28	0.0487	**0.0647**	0.0163	0.0166	0.0203	0.029	0.0247	-0.0017	0.005	high
NE30	0.0938	**0.0842**	0.0549	0.0666	0.0734	0.0044	0.0565	0.0666	0.0631	high
NE32	-0.0292	**0.0617**	0.0099	-0.0041	3.00E-04	0.122	0.0192	0.012	-5.00E-04	high
NE36	0.111	**0.0624**	-4.00E-04	0.0017	2.00E-04	0.0706	0.0095	0.0351	0.0064	high
NE1	-0.0949	**-0.0584**	-0.0231	-0.0318	-0.0163	0.0125	-0.0049	-0.0104	-0.0411	low
NE12	-0.108	**-0.0637**	-0.0513	-0.0513	-0.0523	-0.189	-0.061	-0.0628	-0.0551	low
NE18	-0.131	**-0.063**	-0.0205	-0.0257	-0.02	0.0541	-0.0292	0.0067	-0.0285	low
NE26	-0.0146	**-0.0547**	-0.0067	-0.0137	-0.0013	-0.0901	-0.0049	-0.0094	-0.053	low
NE35	-0.0374	**-0.0634**	-0.0086	-0.0161	-0.0177	-0.0989	-0.0257	-0.0324	-0.0443	low
NE41	0.0643	**-0.0575**	0.001	-8.00E-04	0.0136	-0.0227	0.0287	0.0313	0.027	low
Corrected p-value[a]	1.39E-01	**4.26E-02**	1.39E-01	1.94E-01	2.62E-01	6.94E-02	2.09E-01	2.62E-01	3.91E-01	

Mg_samples	Ca	Cu	**Mg**	P	K	Se	Na	S	Zn	Group
NE3	0.0956	0.0392	**0.0617**	0.0759	0.072	-0.125	0.0905	0.0795	0.0919	high
NE4	0.0929	0.016	**0.076**	0.0855	0.0904	-0.147	0.0929	0.0635	0.112	high
NE5	0.0925	0.0391	**0.0792**	0.0793	0.0839	-0.067	0.0856	0.0629	0.111	high
NE19	0.108	0.0283	**0.0797**	0.0845	0.083	-0.0695	0.0959	0.099	0.0993	high
NE21	0.0842	0.0402	**0.0765**	0.0816	0.0849	-0.0105	0.0983	0.0685	0.0883	high
NE25	0.0712	-0.0091	**0.0907**	0.0984	0.1	-0.0482	0.119	0.0918	0.121	high
NE10	-0.0543	0.0074	**-0.0347**	-0.038	-0.0372	0.0218	-0.0305	-0.0286	-0.0269	low
NE12	-0.108	-0.0637	**-0.0513**	-0.0513	-0.0523	-0.189	-0.061	-0.0628	-0.0551	low
NE17	-0.0673	0.0222	**-0.0455**	-0.0402	-0.0452	-0.1	-0.0497	-0.0479	-0.0225	low
NE20	-0.0575	-0.0189	**-0.0403**	-0.0458	-0.0395	-0.018	-0.0379	-0.0617	-0.0911	low
NE22	0.0138	-0.0353	**-0.0497**	-0.0521	-0.0579	0.0074	-0.015	-0.0233	-0.011	low
NE27	-0.109	-0.0268	**-0.0396**	-0.0458	-0.0366	-0.163	-0.0528	-0.0353	-0.037	low
Corrected p-value[a]	3.07E-04	1.75E-02	**3.17E-09**	7.16E-10	1.06E-09	9.20E-01	1.22E-07	2.69E-07	1.69E-05	

P_samples	Ca	Cu	Mg	**P**	K	Se	Na	S	Zn	Group
NE4	0.0929	0.016	0.076	**0.0855**	0.0904	-0.147	0.0929	0.0635	0.112	high
NE5	0.0925	0.0391	0.0792	**0.0793**	0.0839	-0.067	0.0856	0.0629	0.111	high
NE7	0.138	0.0125	0.0598	**0.0765**	0.0813	-0.152	0.0719	0.0815	0.134	high
NE19	0.108	0.0283	0.0797	**0.0845**	0.083	-0.0695	0.0959	0.099	0.0993	high
NE21	0.0842	0.0402	0.0765	**0.0816**	0.0849	-0.0105	0.0983	0.0685	0.0883	high
NE25	0.0712	-0.0091	0.0907	**0.0984**	0.1	-0.0482	0.119	0.0918	0.121	high
NE12	-0.108	-0.0637	-0.0513	**-0.0513**	-0.0523	-0.189	-0.061	-0.0628	-0.0551	low
NE17	-0.0673	0.0222	-0.0455	**-0.0402**	-0.0452	-0.1	-0.0497	-0.0479	-0.0225	low
NE20	-0.0575	-0.0189	-0.0403	**-0.0458**	-0.0395	-0.018	-0.0379	-0.0617	-0.0911	low
NE22	0.0138	-0.0353	-0.0497	**-0.0521**	-0.0579	0.0074	-0.015	-0.0233	-0.011	low
NE27	-0.109	-0.0268	-0.0396	**-0.0458**	-0.0366	-0.163	-0.0528	-0.0353	-0.037	low
NE34	-0.0422	0.0013	-0.0298	**-0.0406**	-0.0363	-0.0114	-0.0207	-0.0169	-0.0369	low
Corrected p-value[a]	1.22E-04	2.18E-02	3.48E-09	**7.50E-10**	7.50E-10	9.37E-01	2.78E-07	9.77E-07	4.59E-06	

K_samples	Ca	Cu	Mg	P	**K**	Se	Na	S	Zn	Group
NE4	0.0929	0.016	0.076	0.0855	**0.0904**	-0.147	0.0929	0.0635	0.112	high
NE5	0.0925	0.0391	0.0792	0.0793	**0.0839**	-0.067	0.0856	0.0629	0.111	high

NE7	0.138	0.0125	0.0598	0.0765	**0.0813**	-0.152	0.0719	0.0815	0.134	high
NE19	0.108	0.0283	0.0797	0.0845	**0.083**	-0.0695	0.0959	0.099	0.0993	high
NE21	0.0842	0.0402	0.0765	0.0816	**0.0849**	-0.0105	0.0983	0.0685	0.0883	high
NE25	0.0712	-0.0091	0.0907	0.0984	**0.1**	-0.0482	0.119	0.0918	0.121	high
NE10	-0.0543	0.0074	-0.0347	-0.038	**-0.0372**	0.0218	-0.0305	-0.0286	-0.0269	low
NE12	-0.108	-0.0637	-0.0513	-0.0513	**-0.0523**	-0.189	-0.061	-0.0628	-0.0551	low
NE17	-0.0673	0.0222	-0.0455	-0.0402	**-0.0452**	-0.1	-0.0497	-0.0479	-0.0225	low
NE20	-0.0575	-0.0189	-0.0403	-0.0458	**-0.0395**	-0.018	-0.0379	-0.0617	-0.0911	low
NE22	0.0138	-0.0353	-0.0497	-0.0521	**-0.0579**	0.0074	-0.015	-0.0233	-0.011	low
NE27	-0.109	-0.0268	-0.0396	-0.0458	**-0.0366**	-0.163	-0.0528	-0.0353	-0.037	low
Corrected p-value[a]	9.78E-05	2.75E-02	8.57E-09	5.59E-10	**5.85E-10**	8.42E-01	1.20E-07	2.65E-07	6.36E-06	

Se_samples	Ca	Cu	Mg	P	K	**Se**	Na	S	Zn	Group
NE9	0.006	-1.00E-04	-0.0165	-0.0247	-0.0291	**0.107**	-0.0275	-0.0333	-0.0384	high
NE16	-0.0256	-0.0179	-0.0102	-0.0199	-0.0242	**0.114**	-0.0246	0.0106	0.0254	high
NE23	-0.0492	0.291	-0.0121	-0.0223	-0.0311	**0.11**	-0.0222	-0.0327	-0.0745	high
NE24	-0.0234	-0.0192	-0.0233	-0.0303	-0.0326	**0.141**	-0.0231	-0.0536	0.0022	high
NE32	-0.0292	0.0617	0.0099	-0.0041	3.00E-04	**0.122**	0.0192	0.012	-5.00E-04	high
NE39	-0.0478	0.0072	0.0165	0.0159	0.0144	**0.0922**	-0.0144	0.0116	0.0297	high
NE4	0.0929	0.016	0.076	0.0855	0.0904	**-0.147**	0.0929	0.0635	0.112	low
NE6	-0.0622	-0.0355	-0.0225	-0.0042	-0.0216	**-0.169**	-0.0167	-0.0162	0.0116	low
NE7	0.138	0.0125	0.0598	0.0765	0.0813	**-0.152**	0.0719	0.0815	0.134	low
NE8	0.0206	0.0121	0.0281	0.0419	0.043	**-0.202**	0.0408	0.0576	0.032	low
NE12	-0.108	-0.0637	-0.0513	-0.0513	-0.0523	**-0.189**	-0.061	-0.0628	-0.0551	low
NE27	-0.109	-0.0268	-0.0396	-0.0458	-0.0366	**-0.163**	-0.0528	-0.0353	-0.037	low
Corrected p-value[a]	6.11E-01	4.53E-01	6.11E-01	4.53E-01	4.53E-01	**4.52E-09**	4.53E-01	4.53E-01	4.53E-01	

Na_samples	Ca	Cu	Mg	P	K	Se	**Na**	S	Zn	Group
NE3	0.0956	0.0392	0.0617	0.0759	0.072	-0.125	**0.0905**	0.0795	0.0919	high
NE4	0.0929	0.016	0.076	0.0855	0.0904	-0.147	**0.0929**	0.0635	0.112	high
NE5	0.0925	0.0391	0.0792	0.0793	0.0839	-0.067	**0.0856**	0.0629	0.111	high
NE19	0.108	0.0283	0.0797	0.0845	0.083	-0.0695	**0.0959**	0.099	0.0993	high
NE21	0.0842	0.0402	0.0765	0.0816	0.0849	-0.0105	**0.0983**	0.0685	0.0883	high
NE25	0.0712	-0.0091	0.0907	0.0984	0.1	-0.0482	**0.119**	0.0918	0.121	high

NE2	-0.0267	0.0408	-0.0332	-0.0385	-0.0237	0.0219	**-0.0398**	-0.0282	-0.0387	low
NE12	-0.108	-0.0637	-0.0513	-0.0513	-0.0523	-0.189	**-0.061**	-0.0628	-0.0551	low
NE14	0.0089	0.034	-0.0312	-0.0302	-0.0249	0.0584	**-0.0459**	-0.0184	-0.01	low
NE17	-0.0673	0.0222	-0.0455	-0.0402	-0.0452	-0.1	**-0.0497**	-0.0479	-0.0225	low
NE20	-0.0575	-0.0189	-0.0403	-0.0458	-0.0395	-0.018	**-0.0379**	-0.0617	-0.0911	low
NE27	-0.109	-0.0268	-0.0396	-0.0458	-0.0366	-0.163	**-0.0528**	-0.0353	-0.037	low
Corrected p-value[a]	4.01E-04	2.00E-01	3.16E-09	5.54E-10	5.92E-09	7.87E-01	**3.16E-09**	5.15E-07	1.32E-05	

S_samples	Ca	Cu	Mg	P	K	Se	Na	**S**	Zn	Group
NE3	0.0956	0.0392	0.0617	0.0759	0.072	-0.125	0.0905	**0.0795**	0.0919	high
NE7	0.138	0.0125	0.0598	0.0765	0.0813	-0.152	0.0719	**0.0815**	0.134	high
NE19	0.108	0.0283	0.0797	0.0845	0.083	-0.0695	0.0959	**0.099**	0.0993	high
NE25	0.0712	-0.0091	0.0907	0.0984	0.1	-0.0482	0.119	**0.0918**	0.121	high
NE29	0.068	-0.0071	0.0166	0.0229	0.0149	0.0757	0.005	**0.0724**	0.0046	high
NE33	0.11	0.0533	0.0595	0.0687	0.0704	-0.072	0.0552	**0.0751**	0.0862	high
NE12	-0.108	-0.0637	-0.0513	-0.0513	-0.0523	-0.189	-0.061	**-0.0628**	-0.0551	low
NE20	-0.0575	-0.0189	-0.0403	-0.0458	-0.0395	-0.018	-0.0379	**-0.0617**	-0.0911	low
NE24	-0.0234	-0.0192	-0.0233	-0.0303	-0.0326	0.141	-0.0231	**-0.0536**	0.0022	low
NE31	0.0301	-0.0372	-0.0166	-0.0298	-0.0317	0.0347	-0.0057	**-0.0662**	-0.0166	low
NE37	-0.0404	-0.0202	-0.012	-0.0156	-0.0205	-0.0078	-0.0362	**-0.0618**	0.0151	low
NE43	-0.0611	0.0263	-0.0148	-0.0154	-0.0322	0.0047	-0.0047	**-0.0599**	0.0067	low
Corrected p-value[a]	3.17E-04	2.86E-02	2.19E-04	1.30E-04	2.89E-04	3.01E-01	1.01E-03	**1.48E-07**	1.55E-03	

Zn_samples	Ca	Cu	Mg	P	K	Se	Na	S	**Zn**	Group
NE3	0.0956	0.0392	0.0617	0.0759	0.072	-0.125	0.0905	0.0795	**0.0919**	high
NE4	0.0929	0.016	0.076	0.0855	0.0904	-0.147	0.0929	0.0635	**0.112**	high
NE5	0.0925	0.0391	0.0792	0.0793	0.0839	-0.067	0.0856	0.0629	**0.111**	high
NE7	0.138	0.0125	0.0598	0.0765	0.0813	-0.152	0.0719	0.0815	**0.134**	high
NE19	0.108	0.0283	0.0797	0.0845	0.083	-0.0695	0.0959	0.099	**0.0993**	high
NE25	0.0712	-0.0091	0.0907	0.0984	0.1	-0.0482	0.119	0.0918	**0.121**	high
NE11	-0.0537	-0.0232	-0.0242	-0.0357	-0.035	0.053	-0.0189	-0.029	**-0.0571**	low
NE13	-0.0392	-0.0217	-0.023	-0.0321	-0.0293	-0.0044	-0.0377	-0.0459	**-0.0834**	low
NE15	0.0084	0.173	-0.0139	-0.0269	-0.0206	0.0867	-0.019	-0.0081	**-0.0629**	low
NE20	-0.0575	-0.0189	-0.0403	-0.0458	-0.0395	-0.018	-0.0379	-0.0617	**-0.0911**	low

NE23	-0.0492	0.291	-0.0121	-0.0223	-0.0311	0.11	-0.0222	-0.0327	**-0.0745**	low
NE38	-0.0821	0.008	0.0011	0.0085	0.004	0.0438	0.0074	0.0042	**-0.0575**	low
Corrected p-value[a]	6.81E-06	4.28E-01	7.61E-07	6.71E-06	7.61E-07	3.84E-04	7.61E-07	1.39E-05	**9.45E-09**	

Supplementary Figure S1. Transcription discovery versus reads sequenced saturation curve.

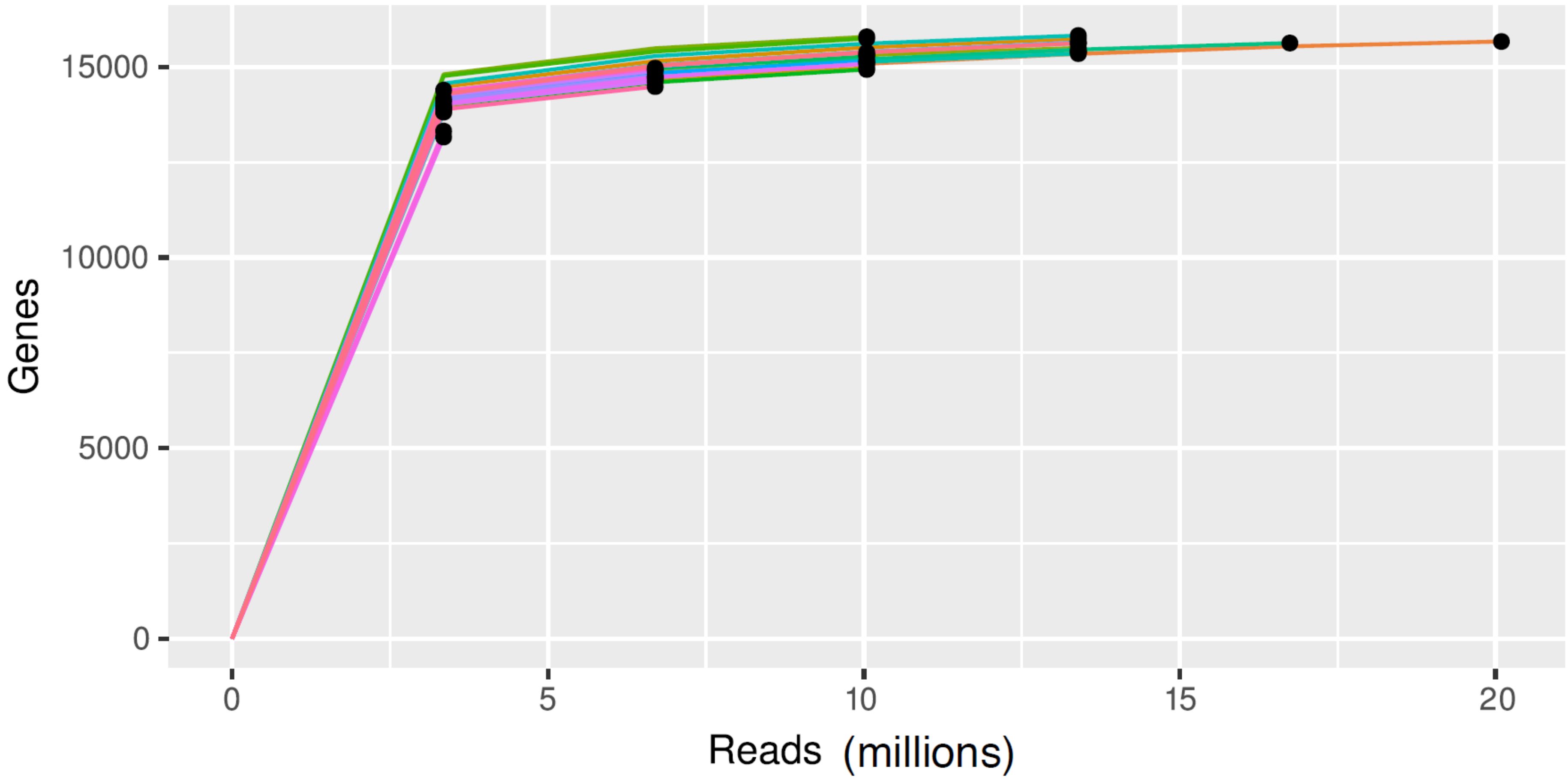

Supplementary Figure S2. Distribution of the samples in the extreme groups regarding all minerals.

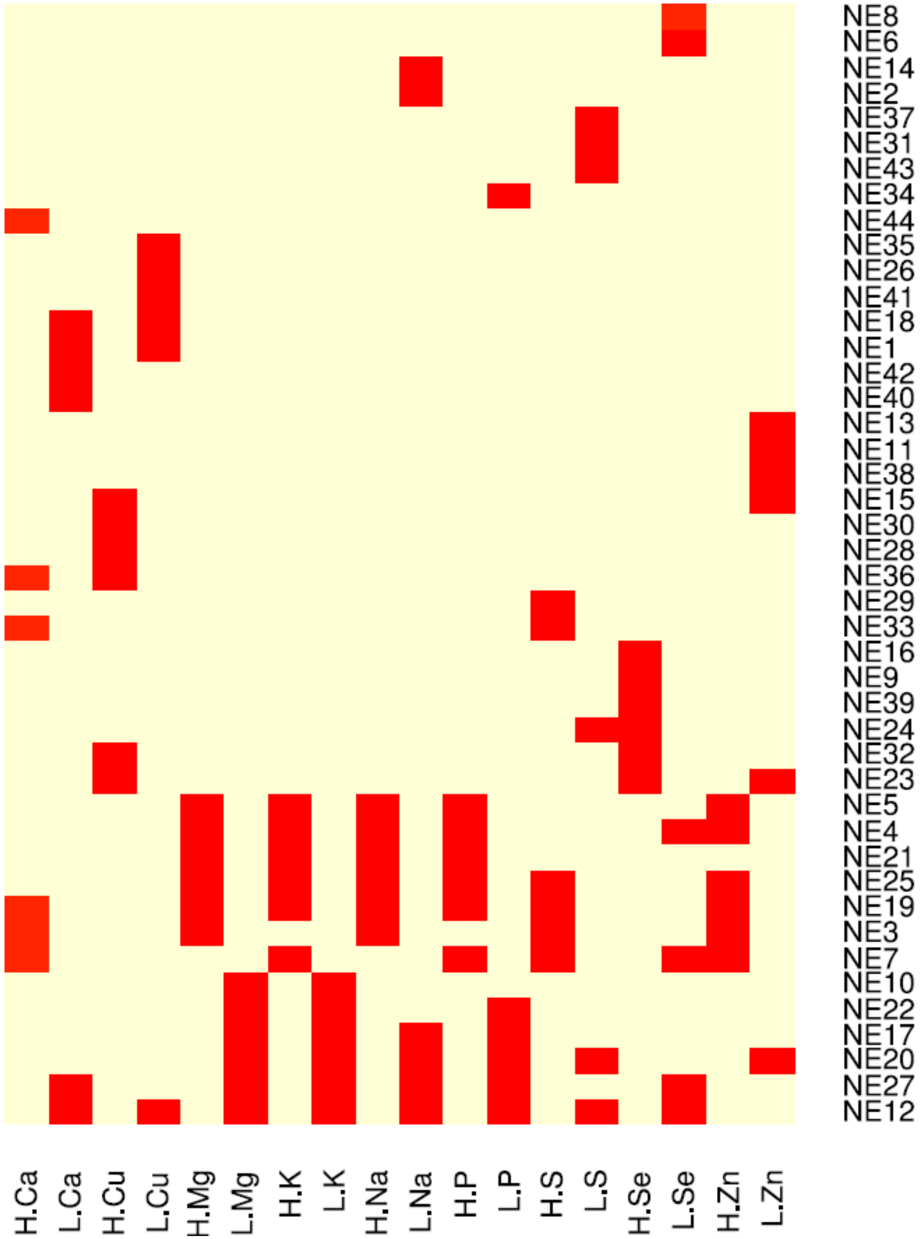

Supplement: Supplementary file 1 — Supplementary information [file 41598_2019_49089_MOESM1_ESM.pdf]
